# Subversion of Lipopolysaccharide Signaling in Gingival Keratinocytes via MCPIP-1 Degradation as a Novel Pathogenic Strategy of Inflammophilic Pathobionts

**DOI:** 10.1128/mBio.00502-21

**Published:** 2021-06-29

**Authors:** Anna Gasiorek, Ewelina Dobosz, Barbara Potempa, Izabela Ciaston, Mateusz Wilamowski, Zuzanna Oruba, Richard J. Lamont, Jolanta Jura, Jan Potempa, Joanna Koziel

**Affiliations:** a Department of Microbiology, Faculty of Biochemistry, Biophysics and Biotechnology, Jagiellonian University, Krakow, Poland; b Department of Oral Immunity and Infectious Diseases, University of Louisville School of Dentistry, University of Louisville, Louisville, Kentucky, USA; c Department of General Biochemistry, Faculty of Biochemistry, Biophysics and Biotechnology, Jagiellonian University, Krakow, Poland; d Chair of Periodontology and Clinical Oral Pathology, Faculty of Medicine, Jagiellonian University Medical College, Krakow, Poland; Tufts University School of Medicine; University of Georgia

**Keywords:** MCPIP-1, *Porphyromonas gingivalis*, gingipains, lipopolysaccharide, periodontitis

## Abstract

Periodontal disease (PD) is an inflammatory disease of the supporting tissues of the teeth that develops in response to formation of a dysbiotic biofilm on the subgingival tooth surface. Although exacerbated inflammation leads to alveolar bone destruction and may cause tooth loss, the molecular basis of PD initiation and progression remains elusive. Control over the inflammatory reaction and return to homeostasis can be efficiently restored by negative regulators of Toll-like receptor (TLR) signaling pathways such as monocyte chemoattractant protein-induced protein 1 (MCPIP-1), which is constitutively expressed in gingival keratinocytes and prevents hyperresponsiveness in the gingiva. Here, we found that inflammophilic periodontal species influence the stability of MCPIP-1, leading to an aggravated response of the epithelium to proinflammatory stimulation. Among enzymes secreted by periodontal species, gingipains—cysteine proteases from Porphyromonas gingivalis—are considered major contributors to the pathogenic potential of bacteria, strongly influencing the components of the innate and adaptive immune system. Gingipain proteolytic activity leads to a rapid degradation of MCPIP-1, exacerbating the inflammatory response induced by endotoxin. Collectively, these results establish a novel mechanism of corruption of inflammatory signaling by periodontal pathogens, indicating new possibilities for treatment of this chronic disease.

## INTRODUCTION

Periodontal disease (PD) is a common bacteria-driven inflammatory disease that erodes the supporting tissues of the tooth. The prevalence of periodontitis reaches up to 30% in the adult population of developed countries (1). It is now generally accepted that change in the composition of the bacterial microbiota on the subgingival tooth surface is the primary event in the etiology of periodontitis. These changes are believed to be initiated by keystone pathogens, which occur in low numbers but have a disproportionate influence on the overall pathogenicity of the microbial community.

The best-characterized periodontal keystone pathogen is Porphyromonas gingivalis, which is a member of a consortium of bacteria strongly associated with advanced PD (2–4). The proliferation of this anaerobic Gram-negative organism in the subgingival biofilm initiates quantitative and qualitative changes in the biofilm microbiome, which becomes dysbiotic. In susceptible hosts, dysbiosis triggers an uncontrolled inflammatory immune response (5) with pathological consequences that include periodontal ligament degradation, alveolar bone resorption, attachment loss, and periodontal pocket formation. Among the most important virulence factors of P. gingivalis are cysteine proteases, gingipains (RgpA, RgpB, and Kgp), which contribute for gaining necessary nutrients, binding to the host tissues, and inactivation of the immune system mechanisms (6). Untreated PD can ultimately lead to tooth loss, which occurs in 8% of patients suffering from the severe form of the disease (7).

Inflammation plays a crucial role in innate immune defense against invading microbes. During infection, the reaction is initiated by recognition of bacterially conserved structural motifs, referred to as pathogen-associated molecular patterns (PAMPs), by specialized pattern recognition receptors (PRRs) on host cells. Control of inflammation requires tight regulation of PRR signaling, especially in tissues such as the gingiva, which are continuously exposed to bacteria. In the oral cavity, this important function relies mainly on gingival keratinocytes, which are equipped with mechanisms that efficiently prevent excess inflammation upon direct contact with bacteria and thus limit the proliferation of periodontal pathogens, which thrive under inflammatory conditions (8).

Among the potent negative regulators of signaling via PRRs is monocyte chemoattractant protein-induced protein 1 (MCPIP-1) (9). This *Zc3h12a* gene product was described in 2006 as a factor induced by MCP-1, a chemoattractant responsible for the recruitment of monocytes and macrophages, and is involved in the pathogenesis of chronic inflammatory conditions (9, 10). MCPIP-1 was described as a negative regulator of bacterial endotoxin signaling and of proinflammatory cytokines, including interleukin 1 beta (IL-1β) and IL-6 (11–13). Further studies revealed that the MCPIP-1 protein contains a zinc finger motif (CCCH) and PilT N-terminal nuclease domain (PIN). The zinc finger domain of MCPIP-1 provides nucleic acid-binding properties, whereas the PIN of the MCPIP-1 domain is responsible for its RNase activity that governs the stability of some cytokine-encoding transcripts (IL-1β, IL-6, IL-8, and IL-12p40) (12, 14, 15). It has been also shown that MCPIP-1 displays deubiquitinase activity and can remove ubiquitin molecules from TRAF2, TRAF3, and TRAF6 proteins (9), thereby hampering the JNK and NF-κB signaling pathways. Such functions of MCPIP-1 support the strong anti-inflammatory properties of this protein and indicate a crucial role in restoring homeostasis in infected tissues.

In the context of periodontal tissue damage driven by inflammation, it is perplexing how little is known about expression and function of inflammation-curbing regulators in the gingiva, especially during progression of periodontal disease (16), which is fueled by inflammophilic P. gingivalis in association with dysbiotic bacteria. Therefore, we tested here the hypothesis that MCPIP-1 is downregulated by P. gingivalis, which gains an advantage from the enhanced inflammatory response, thus leading to the development of periodontitis. We found that in infected keratinocytes, MCPIP-1 was degraded by RgpA gingipain, leading to excessive response of epithelial cells to bacterial endotoxin due to abrogated regulation of TLR signaling. At periodontitis sites, such impairment of the MCPIP-1 regulatory function can promote an exaggerated inflammatory response in the tooth-surrounding tissues and contribute to the progression of PD.

## RESULTS

### MCPIP-1 is degraded in gingiva during periodontitis.

**FIG 1:**
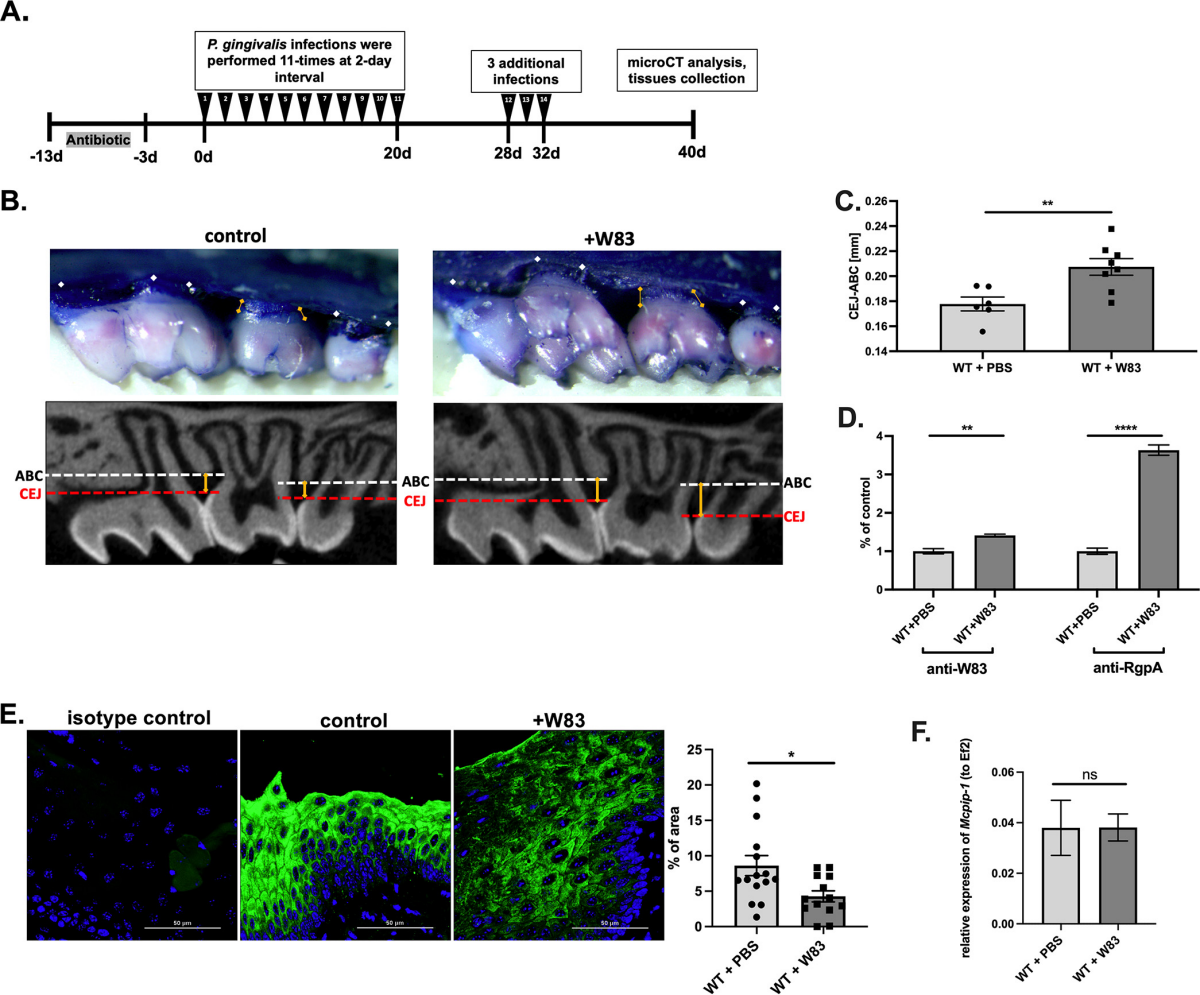
Repeated infection with P. gingivalis W83 induces bone loss in a murine model, contributing to depletion of MCPIP-1 protein in gingiva. (A) Graphical representation of P. gingivalis-induced periodontitis in mice. (B) Visualization of P. gingivalis W83-induced bone loss in wild-type (WT) mice. Representative images of methylene blue (upper) and sectional micro-computed tomography (micro-CT) analysis of gingival tissue (bottom) with indicated cementoenamel junction (CEJ) to alveolar bone crest (ABC) distances on the second molar (yellow lines). (C) Quantitative analysis of the CEJ-ABC distances of the second molar. *n* = 6 in control group; *n* = 8 in infected group (+W83). (D) Levels of anti-W83 and anti-RgpA IgG antibodies in murine sera. Results for the control mice are shown as 1. (E) Visualization of MCPIP-1 protein level (green) by confocal laser scanning microscopy in murine gingiva, along with protein quantification presented as a percentage of area of the fluorescence signal. Analysis performed based on 15 images. (F) Relative expression of *Mcpip-1* transcript in gingival tissue. All data represent mean values ± standard error of the mean (SEM). *, *P* < 0.05; **, *P* < 0.01; ****, *P* < 0.0001; ns, not significant.

To study the role of MCPIP-1 in periodontal disease, we focused on P. gingivalis, a pathogen which is identified in the majority of periodontitis patients (17). In the murine model of periodontitis (Fig. 1A) infection with P. gingivalis caused extensive bone loss (Fig. 1B and C), and chronic infection of periodontium with P. gingivalis (Fig. 1D) induced a significant decrease in MCPIP-1 protein levels (Fig. 1E). Reduction of MCPIP-1 levels is a consequence of posttranslational modifications, as we found that the level of corresponding mRNA was unaffected (Fig. 1F).

### P. gingivalis gingipains effectively degrade MCPIP-1.

**FIG 2:**
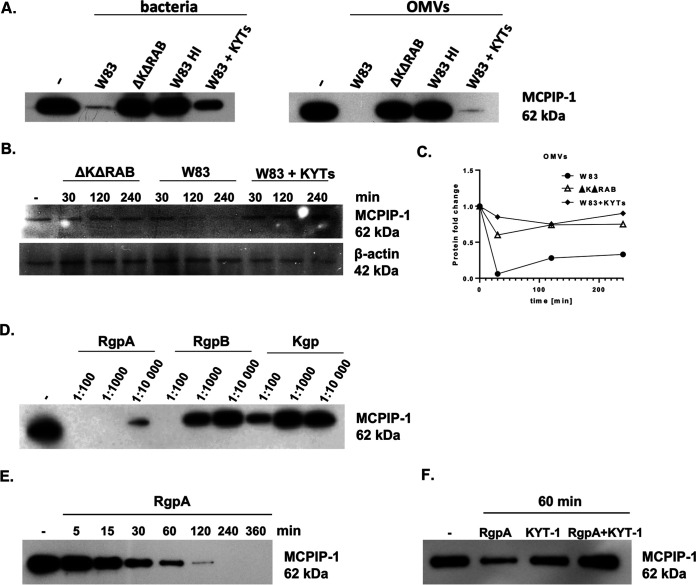
Proteolysis of MCPIP-1 depends on gingipain activity. (A) Recombinant MCPIP-1 protein was incubated with the P. gingivalis wild-type W83 strain, the gingipain-deficient ΔKΔRAB strain, or outer membrane vesicles (OMVs) isolated from both species for 1 h at 37°C. Wild-type bacteria and OMVs were heat-inactivated (HI) at 90°C for 20 min or inactivated with the gingipain-specific inhibitors KYT-1 and KYT-36 (KYTs) for 20 min. (B) Western blot analysis of protein lysates of gingival keratinocytes (TIGKs) infected with P. gingivalis W83 or ΔKΔRAB strains (multiplicity of infection [MOI], 1:100). Gingipain activity was inhibited using a mixture of KYT-1 and KYT-36 inhibitors. (C) Time-dependent MCPIP-1 protein degradation in TIGKs estimated by Western blotting, quantified by densitometry, and presented as a fold change normalized to β-actin levels. Cells were incubated with OMVs with or without KYTs. (D) Recombinant MCPIP-1 protein was incubated for 60 min at 37°C with purified gingipains RgpA, RgpB, or Kgp at indicated molar ratios (gingipain:MCPIP-1) and (E) with RgpA at a 1:10,000 molar ratio for the indicated times. (F) Determination of MCPIP-1 level after exposure of recombinant protein to active RgpA or RgpA inactivated by KYT-1 at a molar ratio of 1:10,000. (A to F) Representative Western blot results are shown.

P. gingivalis is a highly proteolytic pathogen (18). Among the enzymes expressed by this microorganism are gingipains, cysteine proteases that determine 85% of the proteolytic activity of P. gingivalis (19). Therefore, to examine whether gingipains are responsible for MCPIP-1 degradation, we incubated recombinant human MCPIP-1 (rhMCPIP-1) with wild-type P. gingivalis (W83), its isogenic mutant devoid of gingipain activity (ΔKΔRAB), and bacterial cells that were heat-inactivated or pretreated with highly specific inhibitors of gingipain activity, namely KYT-1 and KYT-36 (Fig. 2A). We found that the complete degradation of MCPIP-1 occurs only in the presence of W83 expressing active gingipains (Fig. 2A, left). The same phenomenon was noted when using outer membrane vesicles (OMVs) (Fig. 2A, right), structures enriched in gingipains compared to the amount of gingipains in parent bacterial cells (20). Then, we examined the intracellular proteolysis of MCPIP-1 using human telomerase-immortalized gingival keratinocytes (TIGKs), which are characterized by a constitutively high level of MCPIP-1 (15). Infection of TIGKs with P. gingivalis caused a rapid decrease in the level of MCPIP-1, but only in the presence of bacteria with active gingipains (W83) (Fig. 2B). Degradation of MCPIP-1 was observed at 2 h postinfection and was sustained up to 4 h, while β-actin levels remained unaffected (Fig. 2B). The effect also occurred when TIGKs were exposed to OMVs (Fig. 2C). As we showed that MCPIP-1 degradation occurs during bacterial infection, we aimed to identify the enzyme responsible for the observed phenomenon. For this purpose, we incubated purified arginine- (RgpA and RgpB) and lysine-specific gingipains (Kgp) (21) with rhMCPIP-1 for 1 h at different active enzyme:substrate molar ratios and analyzed samples by SDS-PAGE (Fig. 2D). RgpA was the most efficient gingipain in degradation of rhMCPIP-1. Complete degradation of protein was observed at a ratio of 1:1,000, while upon incubation with RgpB and Kgp at this ratio, the majority of rhMCPIP-1 remained intact (Fig. 2D). rhMCPIP-1 degradation by RgpA occurred in a time-dependent manner, with the majority of the protein degraded after 120 min of incubation at 1:10,000 (Fig. 2E). Finally, we showed inhibition of MCPIP-1 proteolysis when the specific inhibitor of RgpA (KYT-1) was added (Fig. 2F). Collectively, we showed that P. gingivalis infecting keratinocytes degrades MCPIP-1 using predominantly RgpA.

### Internalized RgpA degrades MCPIP-1 in gingival keratinocytes.

**FIG 3:**
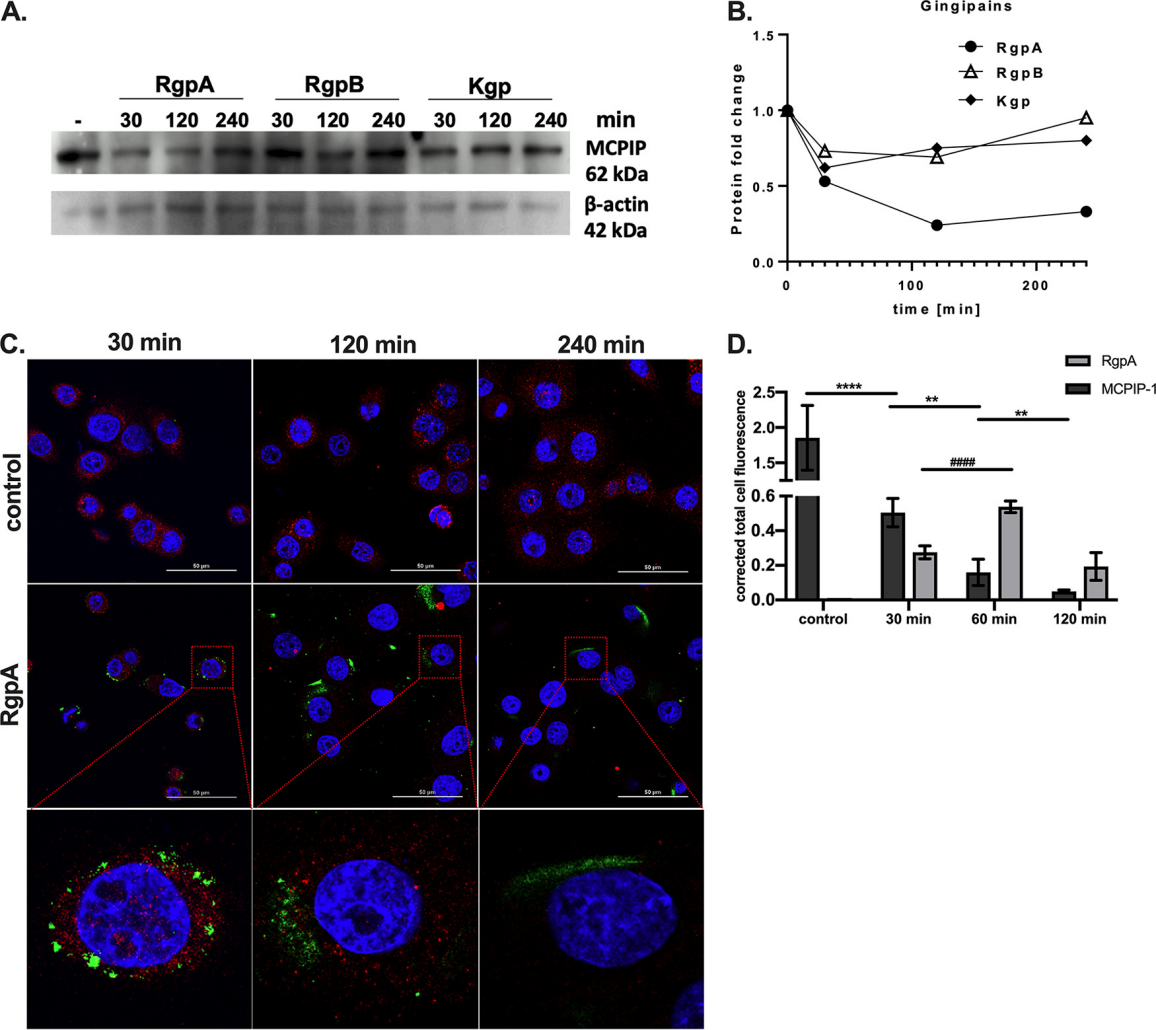
RgpA degrades MCPIP-1 protein in gingival keratinocytes. (A) Representative Western blot analysis, using specific anti-MCPIP-1 antibodies, of protein lysates from TIGK cells treated with 2 nM gingipains (RgpA, RgpB, or Kgp) for the indicated times. (B) Densitometry analysis demonstrating MCPIP-1 protein fold change normalized to β-actin level in TIGK lysates. (C) Visualization of MCPIP-1 protein (red) and RgpA (green) level by confocal laser scanning microscopy in TIGKs. (D) Quantitative analysis of MCPIP-1 and RgpA fluorescence signal. Data show mean fluorescence values (± standard deviation [SD]) in 8 tested fields of view. **, *P* < 0.01; ****, *P* < 0.0001; ####, *P* < 0.0001.

Gingipains are known to penetrate eukaryotic cell membrane and degrade intracellular proteins (22–25). Therefore, we examined the degradation of MCPIP-1 by gingipains in TIGKs. Briefly, TIGKs were exposed to the individual gingipains for different time intervals, and MCPIP-1 protein integrity was evaluated. Western blot analysis revealed MCPIP-1 depletion when TIGKs were preincubated with RgpA, but not with RgpB and Kgp (Fig. 3A and B). Degradation of intracellular MCPIP-1 by RgpA was confirmed by immunofluorescence analysis of TIGKs to visualize MCPIP-1 and internalized RgpA, where the decrease of MCPIP-1 immunostaining intensity coincided with gingipain invasion into the cytoplasm (Fig. 3C and D). Depletion of MCPIP-1 was significant at 30 min post TIGK reaction with 2 nM gingipain (Fig. 3C and D). Taken together, these data suggest that internalized RgpA is directly responsible for MCPIP-1 degradation in the cytoplasm of gingival keratinocytes.

### MCPIP-1 degradation sensitized epithelial cells to endotoxins.

**FIG 4:**
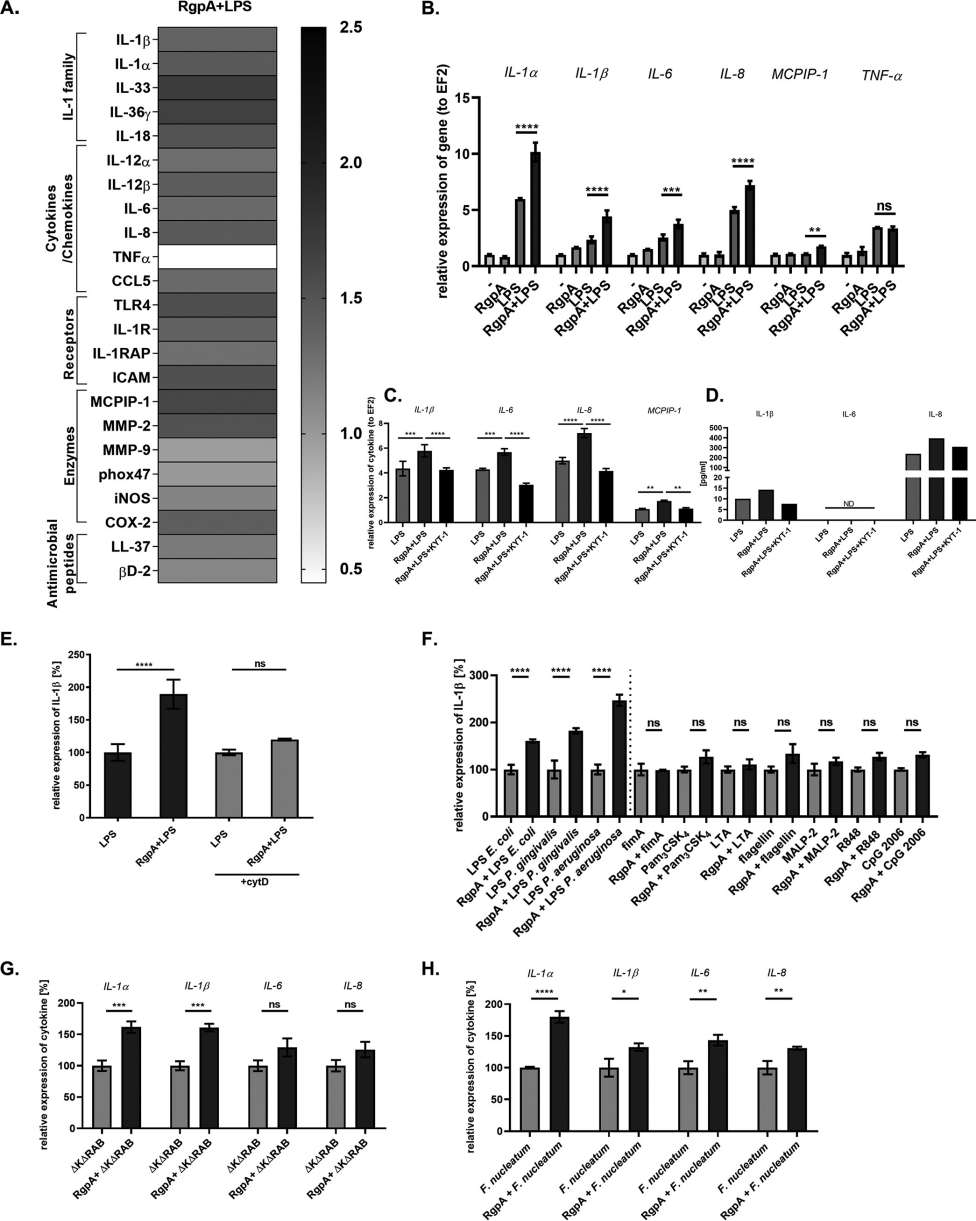
RgpA induces hyperresponsiveness of gingival keratinocytes to endotoxin and Gram-negative bacteria. (A) TIGK cells were prestimulated with 2 nM RgpA for 1 h and treated with lipopolysaccharide (LPS; 20 μg/ml) or infected with bacteria for an additional 3 h. Heat map represents the changes in the expression of genes regulated by LPS signaling in the presence of RgpA. Relative mRNA levels were determined by quantitative reverse transcription-PCR (qRT-PCR). Expression of each gene in cells stimulated only with LPS was established as 1. (B) Quantitative RT-PCR analysis of the proinflammatory cytokines and *MCPIP-1* mRNA level after treatment of cells with RgpA, LPS, and/or RgpA 1 h prior to LPS in comparison to untreated cells. (C, D) Relative expression of *IL-1β*, *IL-6*, *IL-8* and *MCPIP-1* at the mRNA and protein level in TIGK cells. Gingival keratinocytes were pretreated for 1 h with RgpA, and then the specific-RgpA inhibitor KYT-1 was added for 30 min. Subsequently, cells were stimulated with LPS for 3 h. ND, not detected. (E) Relative expression of *IL-1β* after treatment of TIGK cells with cytochalasin D (+cytD) for 30 min prior to their stimulation with LPS and RgpA/LPS in comparison to cytochalasin D-untreated cells. LPS-stimulated cells are presented as 100%. (F) Comparison of *IL-1β* mRNA expression determined by qRT-PCR after a 3-h reaction of TIGKs to different Toll-like receptor (TLR) agonists in comparison to cells additionally pretreated with RgpA for 1 h. TIGKs stimulated with corresponding agonists are presented as 100%. (G, H) Gingiva keratinocytes were preincubated for 1 h with RgpA (2 nM) and then exposed for 3 h to (G) the P. gingivalis gingipain-deficient ΔKΔRAB strain at an MOI of 1:25, or (H) F. nucleatum at an MOI of 1:10. The level of proinflammatory cytokines was determined by qRT-PCR. TIGKs stimulated with bacteria are presented as 100%. Data represent mean values from three independent experiments ± SD. *, *P* < 0.05; **, *P* < 0.01; ***, *P* < 0.001; ****, *P* < 0.0001; ns, not significant.

**FIG 5:**
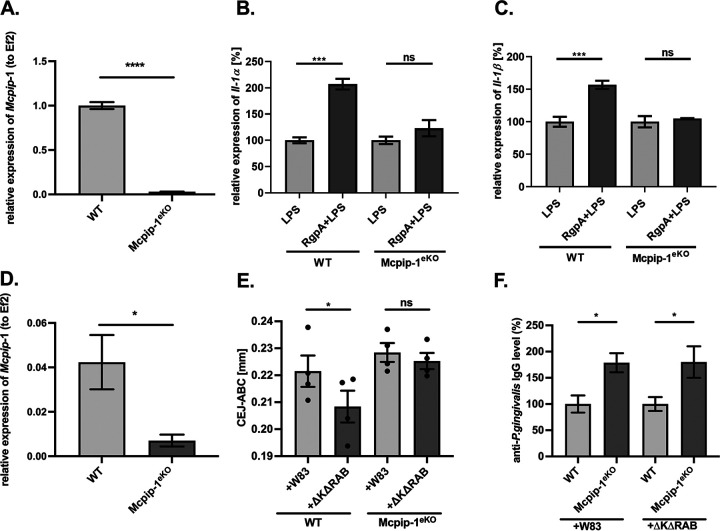
The LPS sensitivity of keratinocytes depends on MCPIP-1 expression. (A) Depletion of *Mcpip-1* in keratinocytes determined by qRT-PCR. (B, C) Wild-type (WT) and *Mcpip-1*-deficient (Mcpip-1^eKO^) murine keratinocytes were exposed to RgpA (2 nM) for 1 h, followed by stimulation with LPS (20 μg/ml) for an additional 3 h. Relative expression of (B) *IL-1α* and (C) *IL-1β* mRNA was determined by qRT-PCR. Cells stimulated with LPS are presented as 100%. (D) Relative expression of *Mcpip-1* transcript in murine gingival tissue (*n* = 4). (E) Quantitative analysis of the CEJ-ABC distances of the second molar of the P. gingivalis-induced bone loss in WT and Mcpip-1^eKO^ mice after repetitive infections with wild-type strain W83 or with the gingipain-deficient ΔKΔRAB mutant (*n* = 4 in each group). (F) Levels of anti-P. gingivalis IgG antibodies in murine sera. The values in WT individuals were defined as 100%. All data represent mean values ± SEM. *, *P* < 0.05; **, *P* < 0.01; ***, *P* < 0.001; ****, *P* < 0.0001; ns, not significant.

Signaling through Toll-like receptors (TLRs) is crucial in the recognition of invading pathogens, although it must be strictly controlled to avoid an excessive inflammatory response (26). MCPIP-1 is one of the major negative regulators of the lipopolysaccharide (LPS)-induced TLR-4 signal transduction pathway (11). As we unambiguously showed that RgpA penetrates the cells and degrades MCPIP-1, we analyzed how depletion of this regulatory protein affects the inflammatory response of RgpA-treated TIKGs to bacterial endotoxin. Human keratinocytes were exposed to RgpA prior to stimulation with bacterial endotoxin, and a transcriptome analysis was performed. This experiment revealed that pretreatment with RgpA resulted in the enhanced response of keratinocytes to LPS compared to that of cells treated with endotoxin alone. RgpA treatment augmented LPS-induced expression of proinflammatory molecules, including cytokines (mostly IL-1 family, IL-6, and IL-8), enzymes (COX-2, MMP-2, and inducible nitric oxide synthase [iNOS]) and receptors (ICAM, TLR-4, and IL-1R) (Fig. 4A). Notably, the observed effect was significant mainly for transcripts regulated by the endoribonuclease activity of MCPIP-1 (IL-1β, IL-6, IL-8, and MCPIP-1) (Fig. 4B) (12, 14, 15, 27). Moreover, applying a selective RgpA inhibitor (KYT-1) showed that stabilization of transcripts was dependent on the enzymatic activity of the gingipain (Fig. 4C). Furthermore, we showed that the elevated level of mRNA due to increased stability of transcripts corresponded to larger amounts of IL-1β and IL-8 protein (Fig. 4D). Using cytochalasin D, we showed that the process of RgpA internalization is necessary for the enhanced response of keratinocytes to LPS (Fig. 4E). Next, we investigated if RgpA can modulate the inflammatory response of keratinocytes to different PAMPs, including LPS from P. gingivalis, Escherichia coli, and Pseudomonas aeruginosa. RgpA treatment significantly enhanced the level of IL-1β transcript in response to endotoxin, regardless of is origin. In stark contrast, RgpA had no effect on the cell response to agonists specific for TLRs other than TLR-4 (Fig. 4F). This observation was confirmed using the model of keratinocytes infected with Gram-negative periodontal pathogens. We found that the P. gingivalis mutant devoid of gingipain activity (the ΔKΔRAB mutant) or Fusobacterium nucleatum induced an accelerated response of epithelial cells when pretreated with RgpA (Fig. 4G and H). Cumulatively, our data show that the intracellular activity of RgpA targeting MCPIP-1 is responsible for the enhanced response of keratinocytes to LPS and Gram-negative pathogens. To further evaluate if MCPIP-1 degradation by RgpA is the essential event in the observed hypersensitivity of keratinocytes to endotoxin, we used epithelial cells with a genetic deficiency of *Mcpip-1*. For that purpose, we isolated cells from Cre-*loxP* mice with tissue-specific silenced *Mcpip-1* in keratinocytes (Fig. 5A), and the results revealed that their exposure to RgpA did not further influence cellular response to endotoxin (Fig. 5B and C). This is consistent with the stronger tissue-damaging inflammatory response *in vivo* of mice deficient in *Mcpip-1* in keratinocytes (Mcpip-1^eKO^; Fig. 5D) and orally infected with P. gingivalis compared to that in wild-type (WT) animals treated in the same way (Fig. 5E). In addition to the increased bone loss, we observed an elevated systemic response, manifested by higher levels of antibodies against P. gingivalis in mice with *Mcpip-1* deficiency (Fig. 5F). Taken together, we confirmed that MCPIP-1 plays an important anti-inflammatory role in the gingiva and limits sensitivity of keratinocytes to LPS-induced inflammatory reactions.

### Protease-rich periodontal pathogens are responsible for the degradation of MCPIP-1 protein.

**FIG 6:**
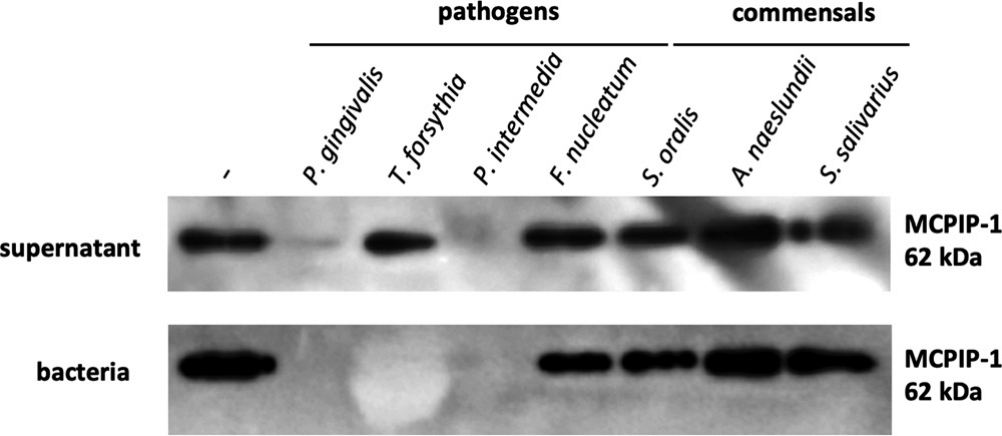
Highly proteolytically active periodontal bacteria degrade the MCPIP-1 protein. Representative Western blot of recombinant human MCPIP-1 protein incubated for 1 h with bacterial supernatants (upper) or bacteria (bottom) classified as pathogens (Porphyromonas gingivalis, Tannerella forsythia, Prevotella intermedia, or Fusobacterium nucleatum) or commensals (Streptococcus oralis, Actinomyces naeslundii, or Streptococcus salivarius).

We examined if periodontal pathogens with documented proteolytic activity (28), other than P. gingivalis, could promote inflammatory reactions via MCPIP-1 modulation. We incubated rhMCPIP-1 with various commensals and pathogens present in the dysbiotic biofilm formed in periodontal pockets. MCPIP-1 was rapidly degraded, visible within 1 h, by species identified as periodontal pathogens, including P. gingivalis, Tannerella forsythia, and Prevotella intermedia (Fig. 6). In contrast, MCPIP-1 was unaffected when incubated with commensals, classified also as early colonizers of the periodontal biofilm. These data suggest that MCPIP-1 degradation is not limited to P. gingivalis and might be considered a general strategy of inflammophilic species.

## DISCUSSION

Periodontitis is a chronic inflammatory disease of the tooth-supporting tissues. The inflammatory milieu of the gingiva favors colonization of the gingival crevice by anaerobic and asaccharolytic bacterial species. These inflammophilic microbes are armed with numerous proteolytic enzymes, which act as highly effective weapons corrupting antimicrobial defense mechanisms, stimulating an excessive response of the host and damage of surrounding tissues, thus providing to the pathogenic community essential proteinaceous nutrients (29). The maintenance of homeostasis in tissues depends on the expression of negative regulators (e.g., SOCS-3 and A20), which dampen immune responses, effectively preventing an uncontrolled inflammatory burst (30, 31). The multifunctional monocyte chemoattractant protein-induced protein 1 (MCPIP-1), which exhibits both endoribonuclease and deubiquitinase activity, thereby inhibiting the NF-κB and JNK signaling pathways, is a primary example of such a regulator (9, 14). Expression of MCPIP-1, like other that of molecules involved in TLR signaling, is regulated at the transcriptional level, and its synthesis is induced by bacteria, fungi, and viral particles (32). Conversely, little is known about posttranslational modifications of MCPIP-1. It has been demonstrated that it can be phosphorylated by the IκB kinase (IKK) and then degraded in the proteasome of primary mouse embryonic fibroblasts (27). Apart from the proteasome, among eukaryotic proteases, only the paracaspase MALT-1 has been described as the protease that targets MCPIP-1 and effectively truncates the MCPIP-1 protein (33). Also, there are no reports that a bacterial protease can affect MCPIP-1 stability. Therefore, it is fascinating that Arg-specific gingipain, a cysteine protease expressed by P. gingivalis, can penetrate cells and efficiently degrade intracellular MCPIP-1, thus impairing the anti-inflammatory function exerted by this important regulator.

The phenomenon of gingipain activity in the intracellular milieu of the host cell was described previously, as extracellularly applied RgpA was capable of entering the nucleus of epithelial cells within 15 min (34). Among intracellular proteins targeted by cell-penetrating gingipains are kinases involved in the tumor necrosis factor alpha (TNF-α)-induced signaling pathway, including mTOR, RIPK1, RIPK2, TAK1, and AKT (22–24). Degradation of these regulators leads to the attenuation of cellular responses. In contrast, proteolysis of the MyD88 adaptor molecule promotes type I interferon expression induced by P. gingivalis (25). Here, we report that MCPIP-1 is yet another intracellular substrate for gingipains. Therefore, we focused on endotoxin signaling in keratinocytes, as MCPIP-1 is classified as the major negative regulator of TLR-4 receptor activation (11, 35).

Gingival keratinocytes are the first line of surveillance and host innate defense against microbes. For efficient detection of invaders, they are equipped with a number of TLR receptors (TLR-1 to TLR-6) sensing many pathogen-associated molecular patterns (PAMPs), including LPS (36). It is of note that large numbers of bacteria in the subgingival biofilm, including periodontitis-associated bacteria such as P. gingivalis, T. forsythia, P. intermedia, and Treponema denticola, are Gram-negative, suggesting persistent stimulation of gingival epithelium by endotoxin. Nevertheless, uncontrolled chronic inflammation apparently develops only in response to a dysbiotic microbiome but not to commensal species. One factor which may explain this paradox, at least partially, is the high constitutive expression of MCPIP-1 in healthy gingiva. High intracellular levels of MCPIP-1 efficiently attenuate the proinflammatory response triggered by LPS recognition by TLR-4 (37). This equilibrium is disturbed when P. gingivalis colonizes the subgingival biofilm in large numbers, and MCPIP-1 is degraded by RgpA. Using an *in vitro* model, we unequivocally showed that MCPIP-1 degradation triggers the enhanced inflammatory response of epithelium to LPS, as manifested by upregulation of expression of a diverse array of proinflammatory mediators. This creates an environment beneficial for P. gingivalis and associated inflammophilic pathobionts, as they are not only resistant to antibacterial components of innate immunity but can also hijack immunological cells, such as neutrophils, for their own benefit (38, 39).

Targeting of MCPIP-1 by RgpA is of high importance, since it provides a plausible clue as to how P. gingivalis with its gingipains can overwhelm checkpoints regulating the immune response of the gingival epithelium. Furthermore, these data shed new light on the cross talk between endotoxins and proteases from periodontal pathogens. Finally, our observation indicates that the role of gingipains in modulation of the host response to LPS is a highly complex phenomenon, as gingipains can also quench the endotoxin signaling in macrophages (40, 41). Sugawara and coworkers described gingipain-dependent attenuation of LPS recognition via degradation of CD14 on the macrophage surface (41). We confirmed this finding (see Fig. S1 in the supplemental material) showing that the level of mRNA encoding proinflammatory cytokines, such as IL-1β, IL-6, and TNF-α, was significantly diminished if human macrophages were preincubated with RgpA prior to LPS treatment. In this respect, our results are groundbreaking and show for the first time the diverse, tissue-specific role of gingipains in the tuning of cellular responses to LPS.

10.1128/mBio.00502-21.1 FIG S1 RgpA reduces the proinflammatory response to endotoxin in human macrophages. The expression of proinflammatory cytokine mRNA was analyzed after initial incubation of human monocyte-derived macrophages (hMDMs) for 1 h with RgpA (2 nM) and further stimulation for an additional 3 h with lipopolysaccharide (LPS; 100 ng/ml). Data represent mean values from three independent experiments ± standard deviation (SD); ****, *P* < 0.0001; ns, not significant. Download FIG S1, TIF file, 0.2 MB. Copyright © 2021 Gasiorek et al. 2021 Gasiorek et al. https://creativecommons.org/licenses/by/4.0/ This content is distributed under the terms of the Creative Commons Attribution 4.0 International license.

**FIG 7:**
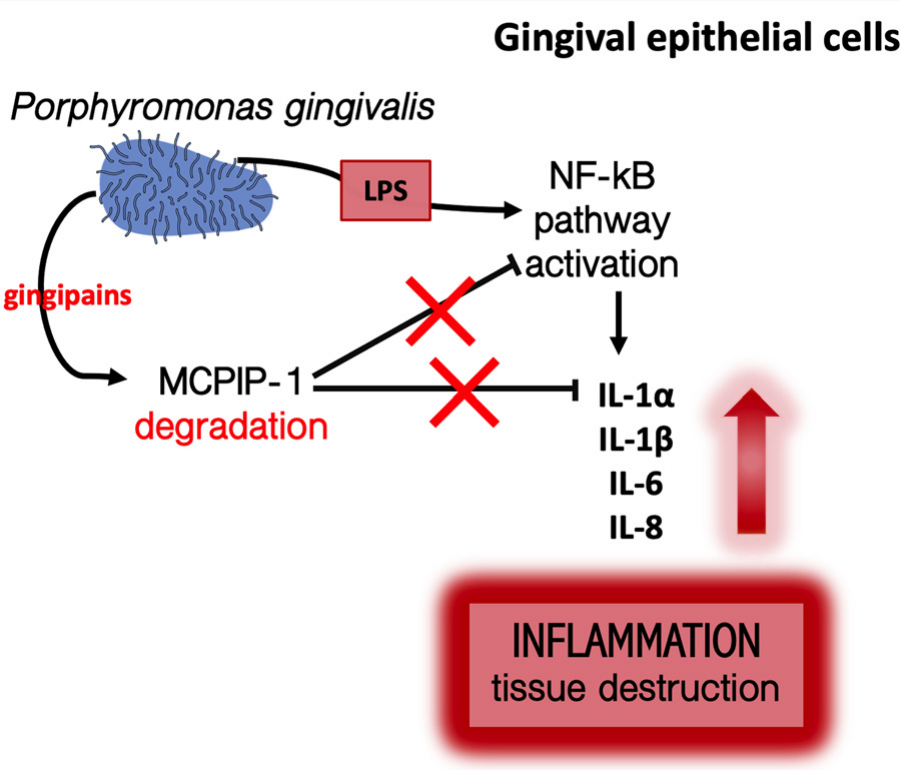
Schematic representation of the putative role of RgpA in the regulation of LPS signaling in gingival keratinocytes. RgpA secreted by P. gingivalis penetrates into the cytoplasm, degrades MCPIP-1, and sensitizes epithelial cells to endotoxins and to both commensal and pathogenic species. Additionally, the released cytokines activate a proinflammatory signaling cascade via the autocrine pathway, leading to exacerbation of the local inflammatory reaction.

An elevated concentration of IL-1 family cytokines was documented in gingival crevicular fluid (GCF) isolated from patients suffering from periodontitis (42–44). The data presented here could be one of the possible explanations of this phenomenon, as we establish significant upregulation of *de novo* synthesis of transcripts encoding IL-1α, IL-β, IL-18, IL-33, and IL-36γ in response to LPS in keratinocytes pretreated with gingipains. These results suggest that gingipains promote accumulation of cytokine proforms followed by the excessive secretion of mature cytokines until exposure of keratinocytes to secondary stimulators, such as peptidoglycans, muramyl dipeptide, or bacterial RNA (45–47), which significantly induce formation of the NRLP3 inflammasome and activation of caspase-1 (48). Moreover, upregulation of the IL-1 family could be of high importance in terms of MCPIP-1 degradation, as interleukin-1β acts via an autocrine or paracrine manner on gingival epithelium, and its proinflammatory signaling is inhibited by MCPIP-1 (12). Our data confirmed this notion, showing an enhanced response of RgpA-pretreated gingival keratinocytes to IL-1β (see Fig. S2 in the supplemental material). Therefore, we propose a model where RgpA degrades MCPIP-1 to induce hyperresponsivity of epithelial cells, primarily to endotoxin released from periodontal pathogens and secondary to cytokines (Fig. 7). It is of note that MCPIP-1 degradation impaired the homeostasis of epithelial tissue, making keratinocytes more reactive even to nonpathogenic bacterial species. We found that MCPIP-1 degradation executed by gingipains can interfere with the cellular response to F. nucleatum and an attenuated mutant of P. gingivalis devoid of gingipain expression (the ΔKΔRAB mutant). The diminished level of MCPIP-1 in keratinocytes predisposes the development of gingival inflammation induced by the ΔKΔRAB mutant. Taken together, MCPIP-1 degradation causes gingipain sensitization of gingival keratinocytes to responses against bacteria, their virulence factors, and inflammatory cytokines.

10.1128/mBio.00502-21.2 FIG S2 RgpA enhances inflammatory response of gingival keratinocytes to interleukin 1 beta (IL-1β). Gingival keratinocytes were preincubated for 1 h with RgpA (2 nM), followed by 3 h of incubation with IL-1β (50 ng/ml). Expression of *IL-6* and *IL-8* mRNA was analyzed. Data represent mean values from three independent experiments ± SD. ****, *P* < 0.0001; ns, not significant. Download FIG S2, TIF file, 0.2 MB. Copyright © 2021 Gasiorek et al. 2021 Gasiorek et al. https://creativecommons.org/licenses/by/4.0/ This content is distributed under the terms of the Creative Commons Attribution 4.0 International license.

The only proven eukaryotic protein that can degrade MCPIP-1 is MALT-1 paracaspase (in complex with Bcl10 and CARD9) (33). It was reported that endotoxin of P. aeruginosa triggers a proinflammatory signal in murine macrophages, which in turn activates Malt-1/Bcl10/Card9 signalosome formation and then MCPIP-1 cleavage (35). We clearly documented in our study that a low dose of RgpA can directly and rapidly degrade MCPIP-1 after 30 min. Moreover, we found unchanged inflammatory responses of RgpA-pretreated cells to LPS when thioridazine, the specific inhibitor of paracaspase, was applied (see Fig. S3 in the supplemental material). However, it is also worth considering the possibility of MALT-1 activation, especially in a long-term infection process, as periodontitis is, which would result in aggravated and prolonged MCPIP-1 depletion. Increased activity of MALT-1 observed in keratinocytes at 4 h and 24 h post P. gingivalis infection (data not shown) supports such a hypothesis. Therefore, one may suppose that the process of MCPIP-1 degradation in periodontitis is multitiered, determined first by gingipain activity and second by MALT-1.

10.1128/mBio.00502-21.3 FIG S3 The proinflammatory response to RgpA is independent of MALT-1 activity. Relative expression of *IL-1β* in telomerase-immortalized gingival keratinocyte (TIGK) cells after their treatment with the MALT-1 inhibitor thioridazine for 30 min prior to their stimulation with RgpA, LPS, or RgpA/LPS in comparison to that in thioridazine-untreated cells. Data represent mean values from three independent experiments ± SD. ***, *P* < 0.001. Download FIG S3, TIF file, 0.2 MB. Copyright © 2021 Gasiorek et al. 2021 Gasiorek et al. https://creativecommons.org/licenses/by/4.0/ This content is distributed under the terms of the Creative Commons Attribution 4.0 International license.

To conclude, this study revealed that MCPIP-1 plays an important role in maintaining homeostasis in the gingival epithelium and highlights a novel mechanism by which periodontal pathogens, such as P. gingivalis, can alter the innate immune response of oral keratinocytes. LPS released by Gram-negative bacteria in the subgingival biofilm, regardless of pathogenicity, triggers a proinflammatory response. This is potentiated by gingipains, which penetrate into gingival cells, leading to MCPIP-1 depletion. The reduced level of MCPIP-1 contributes to the overactivation of the NF-κB signaling pathway, which results in an increase of transcripts encoding proinflammatory cytokines. Subsequently, there is uncontrolled chronic inflammation, periodontal tissue damage, and progression of periodontitis. Taken together, these findings highlight a novel mechanism of corruption of LPS signaling, revealing a new perspective for potential treatment of periodontitis.

## MATERIALS AND METHODS

### Reagents.

Tryptic soy broth (TSB), *N*-acetylmuramic acid, TRI Reagent, l-benzoyl-Arg-*p*NA (l-BA*p*NA), and Tos-Gly-Pro-Lys-*p*NA (Tos-GPK-*p*NA) substrates, gentamicin, LPS from Escherichia coli O111:B4, lipoteichoic acid (LTA), and carboxymethylcelullose were from Sigma-Aldrich. Yeast extract, l-cysteine, hemin, and bovine serum albumin (BSA) were from BioShop Canada, Inc. Dulbecco’s phosphate-buffered saline without Ca^2+^ and Mg^2+^ (PBS), fetal bovine serum (FBS), RPMI 1640, the BCA protein assay kit, reducing sample buffer, Hoechst 33342 stain, eosin, and decalcifier were from Thermo Fisher Scientific. Menadione was from ICN Biomedicals, KYT-1 and KYT-36 from PeptaNova, and saponin from Serva Electrophoresis GmbH. Toll-like receptor agonists were obtained as follows: Pam_3_CSK_4,_ flagellin, and R848 from Enzo Life Sciences, macrophage-activating lipopeptide (MALP-2) from Imgenex, CpG ODN 2006 from Hycult Biotech, and human recombinant IL-1β from BioLegend. Their purity was estimated for CpG 85% (high-performance liquid chromatograph [HPLC]), flagellin 95% (SDS-PAGE), LPS 80%, MALP-2 95% (HPLC), and LTA 97%, according to the manufacturer’s statement. All agonists, except LPS, were endotoxin free.

### Bacterial culture.

The P. gingivalis wild-type strain W83 and the gingipain-deficient ΔKΔRAB strain were grown in TSB medium supplemented with yeast extract, l-cysteine (50 μg/ml), hemin (5 μg/ml), and menadione (0.5 μg/ml). Tannerella forsythia ATCC 43037 was cultured in TSB medium containing hemin (5 μg/ml), *N*-acetylmuramic acid (10 mg/ml), and 10% FBS. Prevotella intermedia 17 and Fusobacterium nucleatum ATCC 10953 were cultivated in TSB medium with yeast extract. All strains mentioned above were cultured under anaerobic conditions (90% N_2_, 5% H_2_, and 5% CO_2_) at 37°C. Streptococcus oralis ATCC 35037, Actinomyces naeslundii ATCC 12104, and Streptococcus salivarius ATCC 7073 were cultured in TSB medium in an atmosphere of 5% CO_2_ at 37°C. All bacterial strains from an overnight culture were centrifuged (5,000 × *g*, 10 min). The bacterial pellet was washed three times in PBS and resuspended in PBS at a final optical density at 600 nm (OD_600_) of 1.

### Animal procedures.

Experiments were conducted using the C57BL/6 murine strain. The Cre-*loxP* system was applied to generate Krt14^Cre^Mcpip-1^fl/fl^ (referred to here as Mcpip-1^eKO^) mice (49). Control Mcpip-1^fl/fl^ animals were designated the WT. Mice were housed in individually ventilated cages in a specific pathogen-free environment and fed with a standard laboratory food and water *ad libitum*. All animals were maintained under controlled environmental conditions (12-h light/12-h dark cycle at 22°C and 60% relative humidity) in the animal care facility at the Faculty of Biochemistry, Biophysics and Biotechnology (Jagiellonian University, Krakow, Poland). All animal procedures were in accordance with the Directive of The European Parliament on the protection of animals used for scientific purposes (2010/63/EU of European Parliament) and were approved by the Local Ethics Committee (Jagiellonian University, Krakow, Poland; permit number 255/2019).

Six- to eight-week-old female mice were administered antibiotics (sulfamethoxazole at 860 μg/ml and trimethoprim at 172 μg/ml; Roche) in water *ad libitum* for 10 days, followed by 3 days without antibiotics. Periodontitis was induced via oral gavage. Briefly, mice were treated with a 100-μl bacterial suspension (1 × 10^9^ CFU of P. gingivalis in 2% carboxymethylcellulose dissolved in PBS) or 2% carboxymethylcellulose (control group) using a stainless-steel feeding needle with olive tip (AgnTho’s). Infections were performed every other day 14 times, with a 7-day break after 11th treatment. One week after the last infection, mice were subjected to microcomputed tomography (micro-CT) measurement, and blood and gingival tissues were harvested for further analyses.

### Quantification of bone loss.

To quantify alveolar bone loss, we used microcomputed tomography (MILabs, The Netherlands) on live animals. The measurement was carried out with the following settings: voltage, 50 kV; tube current, 0.21 mA; exposure time, 75 ms; and step angle, 0.1°. Briefly, the linear measurements of the distances between cementoenamel junction (CEJ) and alveolar bone crest (ABC) were taken at two sites of second molars in the right and left dental arches (50). Data were quantified using PMOD software (PMOD Technologies Ltd., Switzerland). Additionally, bone loss was visualized by methylene blue and eosin staining after euthanasia, as described previously (51). Soft tissue was removed from bones, and the jaws were immersed overnight in 3% hydrogen peroxide. The next day, jaws were incubated in 1% bleach and then washed and air dried. Alveolar bone and teeth were stained with 0.5% eosin for 5 min and 1% methylene blue (Merck Millipore) for 1 min, followed by several washes in distilled water. Bone loss was visualized with a dissecting microscope (SZX-9; Olympus) connected to a digital camera (EOS 1000D; Canon).

### Gingival tissue preparation.

Upper gingiva were resected as described previously (52). Gingival tissue with alveolar bone and teeth was fixed in 4% formaldehyde solution at 4°C for 16 h. After incubation in decalcifier for 2 weeks, tissue was washed 2 times in PBS and dehydrated with graded series of alcohol (from 70% to 100%) and incubated for 1 min in acetone and twice for 5 min in xylene. Gingiva were embedded in melted paraffin, cut to 5-μm sections by a microtome, and mounted on a microscope slide. Before staining, slides were deparaffinized in xylene, rehydrated in a series of alcohol (from 100% to 70%), and boiled for 20 min in sodium citrate buffer (10 mM sodium citrate and 0.05% Tween 20 [pH 6.0]). Prepared slides were stored for further analyses.

### IgG level in serum.

To determine IgG-specific antibody in murine sera, the enzyme-limited immunosorbent assay (ELISA) method was performed. Briefly, the wells were coated with 250 ng purified RgpA in carbonate-bicarbonate buffer (pH 9.5) for 16 h at 4°C or with 100 μl of formaldehyde-fixed *P. gingivalis* W83 cells (OD_600_ = 0.5) for 1 h at 37°C and then for 16 h at 4°C. The next day, wells were washed 3 times with PBS and blocked in 2% BSA in PBS. After several washes, 4-fold dilutions of murine sera were added to the wells and incubated for 1 h in 37°C. Following washing 3 times with PBS, sheep anti-mouse IgG peroxidase-conjugated antibody (Sigma-Aldrich; 1:4,000 diluted in 1% BSA in PBS) was added for 1 h at 37°C. Wells were washed 5 times, and 3,3,5,5-tetramethylbenzidine substrate (TMB) was added (BD Biosciences). The reaction was stopped with 2N sulfuric acid, and the color intensity was recorded at 450 nm.

### Cell culture.

Telomerase-immortalized gingival keratinocytes (TIGKs) (36), derived from primary gingival epithelial cells (GECs), and murine primary keratinocytes, isolated from newborn skin as described previously (49), were routinely cultured at 37°C and 5% CO_2_ in KBM-Gold keratinocyte basal medium supplemented with Single Quots (Lonza). For experiments, keratinocytes were seeded on 12-well plates (TPP) in medium without antibiotics. Human monocyte-derived macrophages (hMDMs) differentiated from peripheral blood mononuclear cells (PBMCs) were isolated as described previously (53), seeded on 24-well plates in RPMI 1640 medium supplemented with 10% heat-inactivated autologous human plasma and 50 μg/ml gentamicin, and cultivated under standard conditions (37°C and 5% CO_2_). Blood was purchased from the Red Cross (Krakow, Poland), which deidentifies blood materials as appropriate for human subject confidentiality assurances. Thus, this study adheres to appropriate exclusions from human subject approval.

### Isolation of P. gingivalis outer membrane vesicles.

Isolation of OMVs from P. gingivalis was performed as described recently (38). Bacteria (OD_600_ = 1) were sonicated for 90 s in a water bath to release OMVs from the cell surface. Intact bacteria were removed by centrifugation (10,000 × *g*, 20 min, 4°C), and supernatant was subjected to ultracentrifugation (150,000 × *g*, 1 h, 4°C). The pellets containing OMVs were resuspended in buffer (20 mM BisTris, 150 mM NaCl, 5 mM CaCl_2_ [pH 6.8]) and analyzed for protein concentration using a bicinchoninic acid (BCA) assay.

### Gingipains.

Arg- and Lys-specific gingipains RgpA/RgpB and Kgp were purified from spent growth medium of P. gingivalis HG66, as described previously (54, 55). To determine the concentrations of active gingipains, active-site titration was performed. Gingipains were titrated using specific inhibitors, namely, KYT-1 (for Rgp) or KYT-36 (Kgp) (56). For each experiment, enzymes were activated with 20 mM l-cysteine for 15 min at 37°C in TNC buffer (100 mM Tris, 150 mM NaCl, and 5 mM CaCl_2_ [pH 7.5]). For inactivation, proteases were incubated for 15 min at 37°C with 1 μM KYT-1 and KYT-36. The efficiency of enzyme inhibition was verified using l-BA*p*NA as a substrate for Rgps and Tos-GPK-*p*NA for Kgp.

### Interaction of keratinocytes to gingipains.

Keratinocytes were infected for 30 min, 2 h, and 4 h with P. gingivalis W83 or ΔKΔRAB strains at a multiplicity of infection (MOI) of 1:100 in the presence or absence of KYT-1 and KYT-36 inhibitors (final concentration, 1 μM each) or treated with 2 nM gingipains with or without 1 μM KYT-1 (RgpA and RgpB) and/or KYT-36 (Kgp). After the indicated times, cells were lysed in radioimmunoprecipitation assay (RIPA) buffer (0.25% Na deoxycholate, 0.05% SDS, 0.5% Nonidet P-40, 2.5 mM EDTA, and protease inhibitor cocktail) for protein isolation, and lysates were subjected to SDS-PAGE. For confocal fluorescence microscopy, TIGKs (0.05 × 10^6^ cells) were seeded on coverslips in KBM-Gold keratinocyte basal medium and incubated overnight at 37°C and 5% CO_2_. Cells were incubated with 2 nM RgpA for 30 min, 1 h, and 2 h, then washed with PBS and fixed with 3.7% formaldehyde in PBS for 10 min.

### Immunofluorescence staining.

Slides with gingival tissue or with TIGK cells were blocked with PBS containing 5% FBS, 1% BSA, 0.05% Tween 20, and 2 mM EDTA for 1 h at room temperature (RT). Slides were treated with 0.1% saponin in PBS for 30 min at RT and stained with primary antibodies diluted in buffer (3% BSA and 0.1% saponin in PBS). TIGK cells were stained for 1 h at RT with mouse anti-MCPIP-1 (10 μg/ml; R&D) and rabbit anti-RgpA (10 μg/ml) antibodies; gingival tissues were stained with rabbit anti-Mcpip-1 (13.5 μg/ml; GeneTex). Slides were washed with 0.1% saponin in PBS and stained with secondary antibodies for 45 min at RT. All slides were incubated with goat anti-rabbit antibodies conjugated with Alexa 488 (1:500; Cell Signaling); in addition, TIGK cells were stained with goat anti-mouse antibodies conjugated with Alexa 647 (1:500; Cell Signaling). After several washes with 0.1% saponin in PBS, cell nuclei were stained with Hoechst 33342 (1 μg/ml) for 10 min at RT. Then slides were washed in PBS and mounted in fluorescence medium (Dako). Images were captured with a confocal laser scanning microscope (LSM 880; Zeiss). Quantification of fluorescence signal was with ImageJ software and is displayed as corrected total cell fluorescence (CTCF) or as a percentage of area for fluorescence signal.

### Quantitative reverse transcription-PCR.

Total cellular RNA was isolated from TIGKs, hMDMs, and murine keratinocytes using TRI Reagent, while total RNA from murine gingival tissue was isolated using a commercially available kit according to the manufacturer’s instruction (total RNA minikit; A&A). Reverse transcription was performed using a high-capacity cDNA reverse transcription kit (Applied Biosystems). cDNA was synthesized from 800 ng of RNA in a total volume of 20 μl, according to the manufacturer’s instructions. The quantitative PCR was carried out in a final volume of 15 μl, which contained 1 μl of cDNA sample, 10 μM forward and reverse primers, and 1× GoTaq qPCR master mix (Promega). The primers and reaction conditions used in quantitative reverse transcription-PCR (qRT-PCR) are listed in Table S1 in the supplemental material. The PCR was initiated by denaturation for 5 min, and the indicated amplification program was carried out for 40 cycles with a final elongation step at 72°C for 10 min. *EF-2* is a housekeeping gene that used for normalization. The cycle threshold (*C_T_*) values were calculated and analyzed using the ΔΔ*C_T_* method (57). To confirm the specificity of the quantitative PR (qPCR), a melt curve analysis was performed.

10.1128/mBio.00502-21.4 TABLE S1 Oligonucleotides used in the quantitative reverse transcription-PCR (qRT-PCR). ^1^Programs are indicated as follows: 1, denaturation; 2, annealing; 3, elongation. Download Table S1, PDF file, 0.1 MB. Copyright © 2021 Gasiorek et al. 2021 Gasiorek et al. https://creativecommons.org/licenses/by/4.0/ This content is distributed under the terms of the Creative Commons Attribution 4.0 International license.

### Degradation of the rhMCPIP-1 by bacteria, OMVs, and gingipains.

Recombinant human MCPIP-1 (rhMCPIP-1) was obtained according to the procedure described previously (15). Purified protein (100 ng) was incubated for 1 h at 37°C with P. gingivalis W83 or ΔKΔRAB strains, T. forsythia, P. intermedia, F. nucleatum, S. oralis, A. naeslundii, or S. salivarius (2 × 10^7^ CFU suspended in PBS), or with OMVs (70 ng) isolated from P. gingivalis. Activity of P. gingivalis enzymes was inhibited by adding 1 μM KYT-1 and KYT-36. In assays with purified gingipains (RgpA, RgpB, and Kgp), rhMCPIP-1 (100 ng) was incubated in TNC buffer with l-cysteine with or without KYT. The reaction was stopped by adding a reducing sample buffer, followed by a denaturation step for 5 min at 95°C. After determination of protein concentration using a BCA protein assay, samples were subjected to SDS-PAGE.

### Western blot.

Protein samples (20 μg) were loaded to 10% SDS-PAGE gels, separated under reducing conditions, and transferred to polyvinylidene difluoride (PVDF) membranes (Merck Millipore) in transfer buffer (25 mM Tris, 0.2 M glycine, and 20% methanol). Nonspecific binding sites were blocked with 5% skim milk in TBST buffer (20 mM Tris, 0.5 M NaCl, and 0.05% Tween 20 [pH 7.5]) for 2 h at RT and incubated overnight with the following primary antibodies: rabbit anti-MCPIP-1 (Abbiotec), diluted 1:1,000 for cell lysates or 1:5,000 for rhMCPIP-1, and mouse anti-β-actin (BD Bioscience) at 1:10,000 dilution. Membranes were washed in TBST buffer and incubated with horseradish peroxidase (HRP)-conjugated secondary antibodies for 1 h at RT with goat anti-rabbit IgG (Cell Signaling), diluted 1:10,000 for blots of cell lysates or 1:20,000 for blots of rhMCPIP-1, and goat anti-mouse (1:10,000, BD Bioscience). Then, membranes were washed extensively with TBST buffer, developed using Luminata Crescendo substrate (Merck Millipore) and exposed to medical X-ray film (Kodak).

### Stimulation of RgpA-sensitized cells with TLR agonists, IL-1β, and bacteria.

Gingival keratinocytes (0.7 × 10^6^ cells), hMDMs (0.3 × 10^6^ cells), and murine skin keratinocytes (0.35 × 10^6^) were preincubated for 1 h with activated RgpA at a final concentration of 2 nM, followed by reaction of cells with LPS from E. coli (20 μg/ml). In addition, TIGK cells were stimulated with P. gingivalis LPS (Invivogen) (20 μg/ml), P. aeruginosa LPS (isolated from a clinical strain of P. aeruginosa) (20 μg/ml), Pam_3_CSK_4_ (10 μg/ml), LTA (20 μg/ml), flagellin (0.1 μg/ml), MALP-2 (0.1 μg/ml), R848 (10 μM), CpG-B (ODN 2006, 20 μM), FimA purified from ATCC 33277 (80 μg/ml), or recombinant human IL-1β (50 ng/ml). For some experiments, keratinocytes were pretreated with 2 nM active RgpA for 1 h and then further infected with the P. gingivalis ΔKΔRAB strain (MOI, 1:25) or with F. nucleatum ATCC 10953 (MOI, 1:10). Three hours post stimulation with TLR agonists, IL-1β, or bacteria, culture media were collected for determination by ELISA of the level of released cytokines, and cells were lysed for RNA isolation using TRI Reagent.

### ELISA.

The levels of IL-8, IL-6 (BD Biosciences), and IL-1β (BioLegend) were determined using commercially available ELISA kits according to the manufacturer’s instructions. All cytokines were estimated in culture medium, except for IL-1β, which was determined in cell lysate.

### Statistical analysis.

All presented experiments were performed at least in triplicate, and results are expressed as mean ± standard deviation (SD) or mean ± standard error of the mean (SEM). Statistical significance was determined using Student’s *t* test or one- and two-way analysis of variance (ANOVA) followed by Bonferroni *post hoc* tests. A *P* value of <0.05 was considered statistically significant.
