# Supplementary figures and images for: Subversion of Lipopolysaccharide Signaling in Gingival Keratinocytes via MCPIP-1 Degradation as a Novel Pathogenic Strategy of Inflammophilic Pathobionts
Source: mBio. 2021 Jun 29;12(3):e00502-21. doi: 10.1128/mBio.00502-21 (PMC8262937; doi:10.1128/mBio.00502-21)

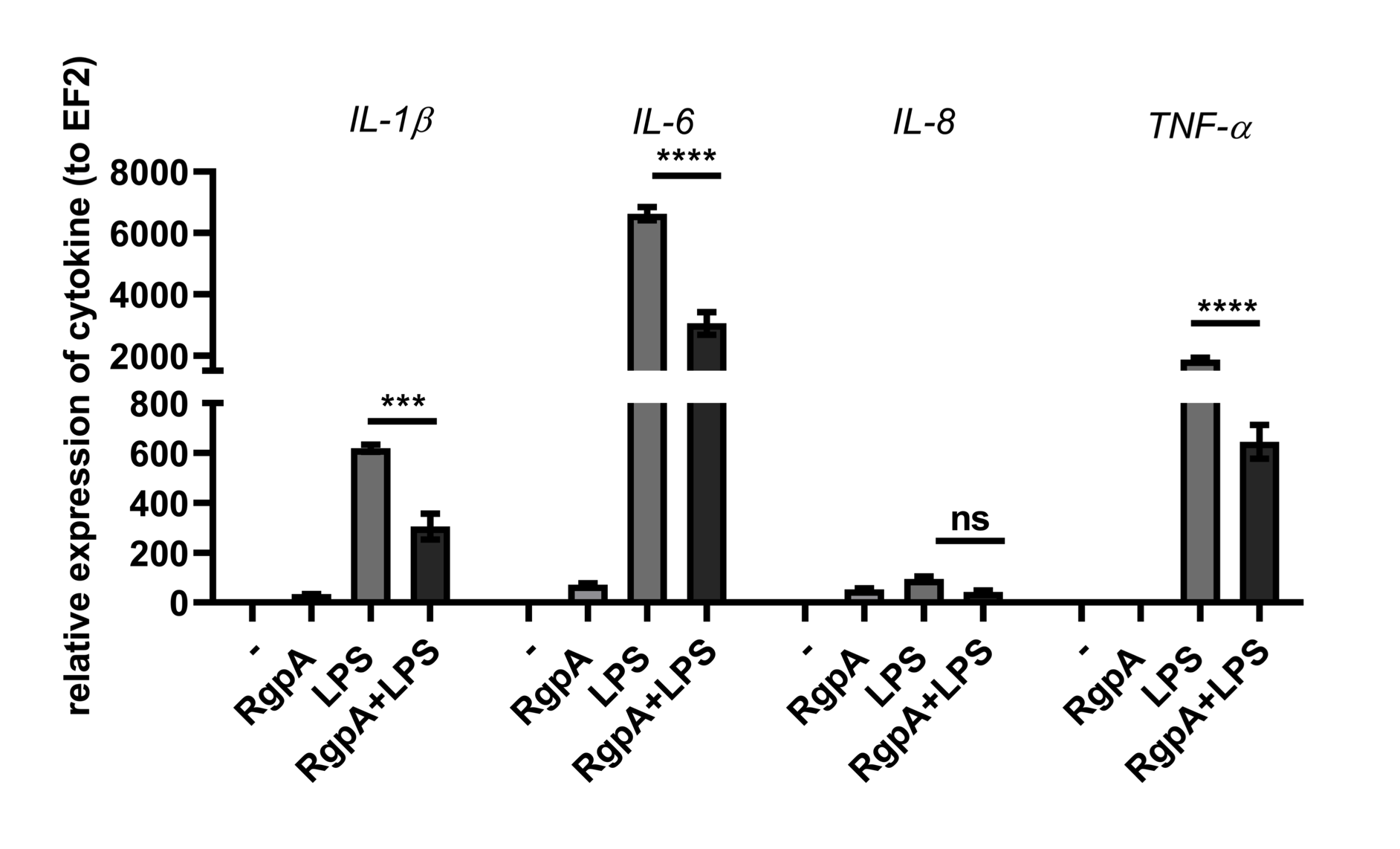

Supplement: FIG S1 [file mbio.00502-21-sf001.tif]

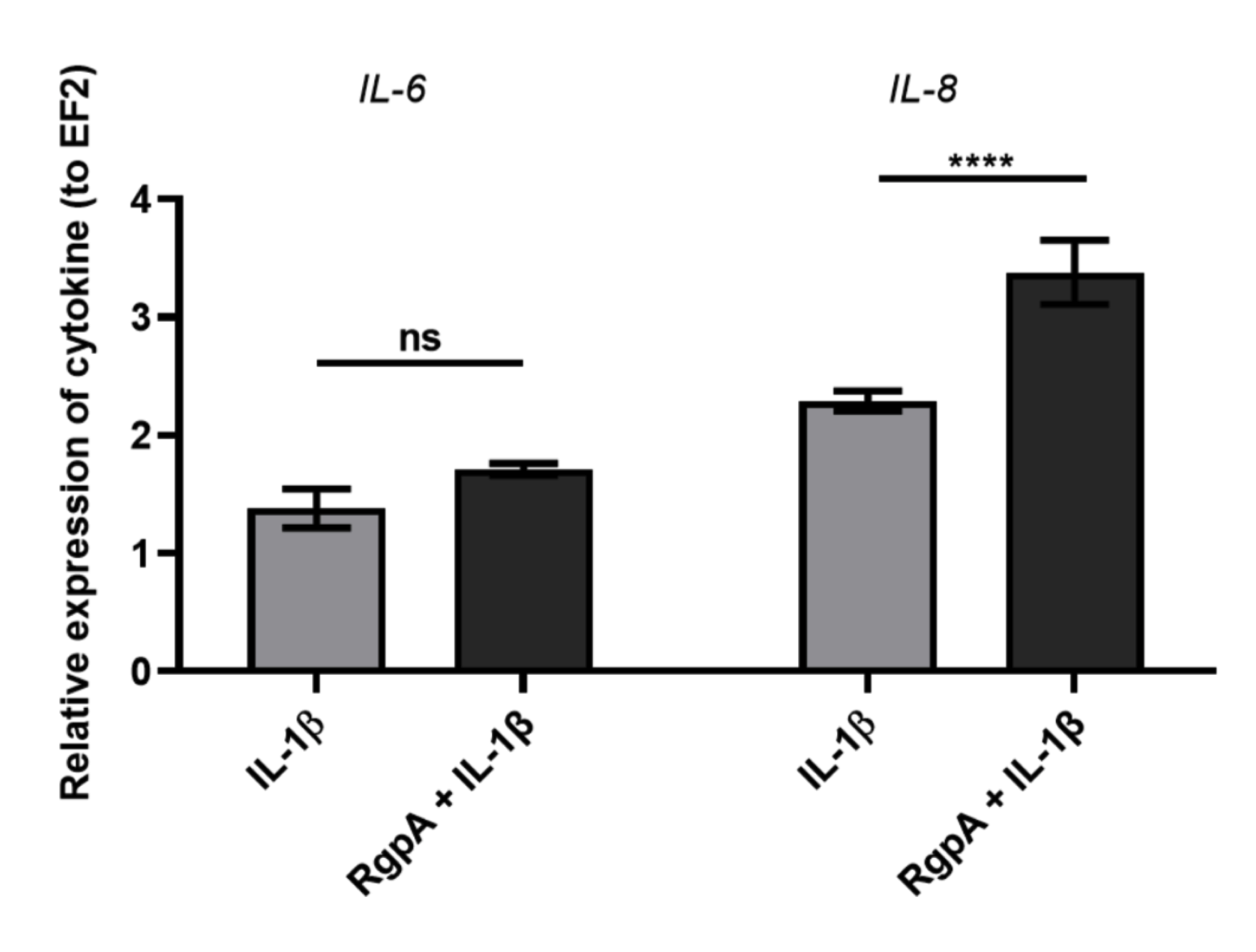

Supplement: FIG S2 [file mbio.00502-21-sf002.tif]

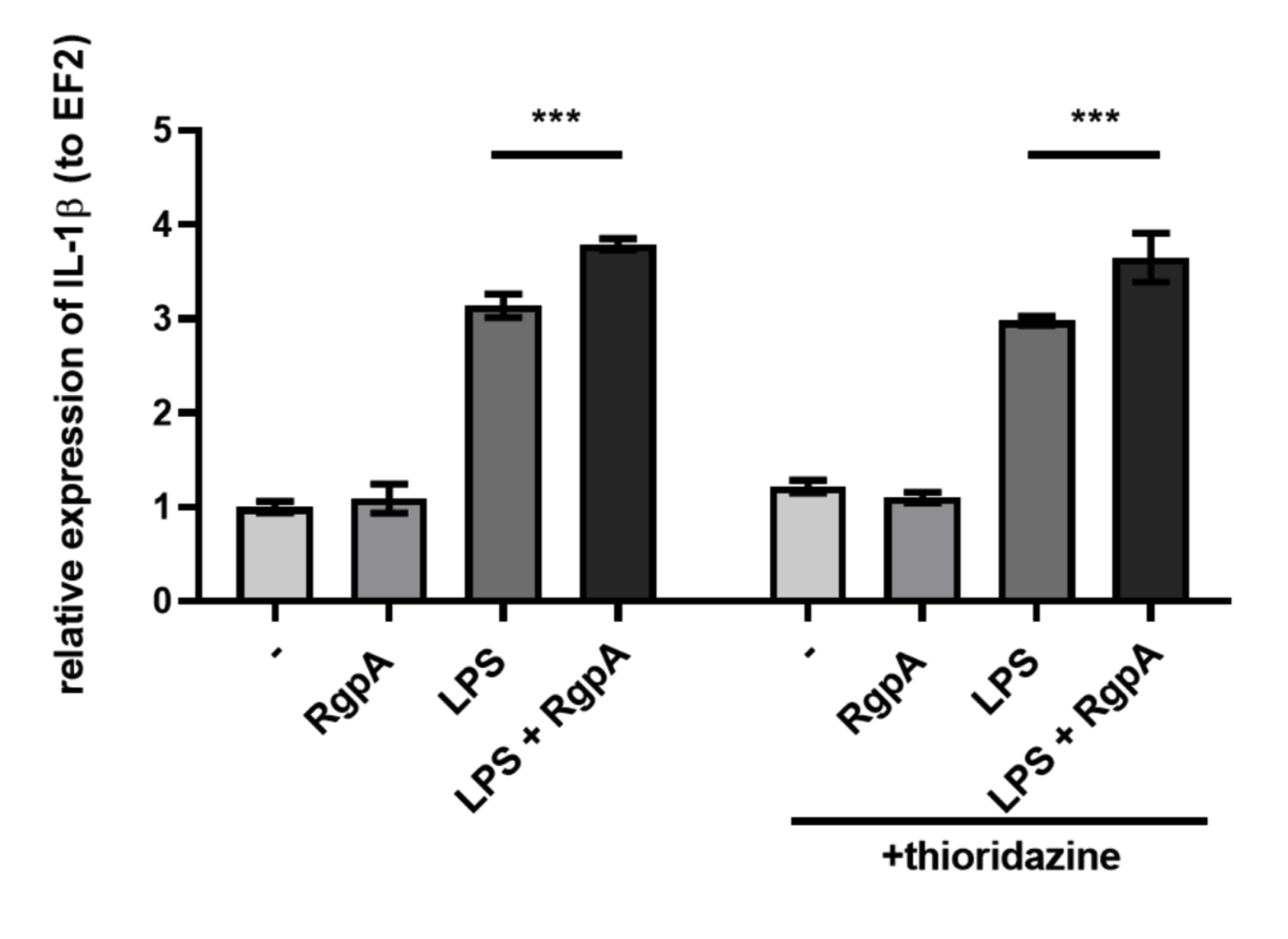

Supplement: FIG S3 [file mbio.00502-21-sf003.tif]
