# Supplementary material for: Subversion of Lipopolysaccharide Signaling in Gingival Keratinocytes via MCPIP-1 Degradation as a Novel Pathogenic Strategy of Inflammophilic Pathobionts
Source: mBio. 2021 Jun 29;12(3):e00502-21. doi: 10.1128/mBio.00502-21 (PMC8262937; doi:10.1128/mBio.00502-21)
Supplement: TABLE S1 [file mbio.00502-21-st001.pdf]

Table S1. Oligonucleotides used in the qRT-PCR. [1] Denaturation (1); Annealing (2); Elongation (3).

**Oligonucleotide**	**Sequence**	**Program**[1]
**Human primers**		
EF-2 F	5'- GACATCACCAAGGGTGTGCAG-3'	
EF-2 R	5'- TTCAGCACACTGGCATAGAGGC-3'	
IL-1α F	5'-CGCCAATGACTCAGAGGAAGA-3'	
IL-1α R	5'-AGGGCGTCATTCAGGATGAA-3'	
IL-1R F	5'-CCTGCTATGATTTTCTCCCAATAAA-3'	
IL-1R R	5' AACACAAAAATATCACAGTCAGAGGTAGAC 3'	
IL-1RAP F	5'-CTTTGACCGAGACAGTCTG-3'	
IL-1RAP R	5'-GTTCCCTGGAGCACGTAGTTG-3'	1. 95˚C, 30 s<br>2. 62˚C, 30 s<br>3. 72˚C, 45 s
MCPIP-1 F	5′-GGAAGCAGCCGTGTCCCTATG-3′	
MCPIP-1 R	5′-TCCAGGCTGCACTGCTCACTC-3′	
NCF-1 F (p47phox)	5'-AGAAGAGCGAGAGCGGTTG-3'	
NCF-1 R (p47phox)	5'-CCAGGAGCTTGTGAATGACCT-3'	
PTGS2 F (COX-2)	5'- TGGTGCCTGGTCTGATGATGTATGC-3'	
PTGS2 R (COX-2)	5'- ATCTGCCTGCTCTGGTCAATGGAA-3'	
CAMP F (LL-37)	5'-ACACAGCAGTCACCAGAGGATTGT-3'	
CAMP R (LL-37)	5'-AAATCACCCAGCAGGGCAAATCTC-3'	
βD-2 F	5'-GATGCCTCTTCCAGGTGTTTTT-3'	
βD-2 R	5'-GGATGACATATGGCTCCACTCTT-3'	
IL-33 F	5'-AGGTGACGGTGTTGATGGTAAG-3'	
IL-33 R	5'-GCATATTATGAAGGACAAAGAAGGC-3'	
IL-36γ F	5'-TAGGACCTCCACCCTTGAGTC-3'	
IL-36γ R	5-AATGATGGGCTGGTCTCTCTT-3'	
IL-18 F	5'-ATCGCTTCCTCTCGCAACAA-3'	
IL-18 R	5'-CTTCTACTGGTTCAGCAGCCATCT-3'	
IL-12α F	5'-ATGATGGCCCTGTGCCTTAG-3'	
IL-12α R	5'-TCCGGTTCTTCAAGGGAGGA-3'	1. 95˚C, 30 s<br>2. 63˚C, 30 s<br>3. 72˚C, 45 s
IL-12β F	5'-ATTCTGCGTTCAGGTCCAGG-3'	
IL-12β R	5'-TCCGGTTCTTCAAGGGAGGA-3'	
CCL5 F	5'-CCTCATTGCTACTGCCCTCT-3'	
CCL5 R	5'-GGTGTGGTGTCCGAGGAATA-3'	
TLR4 F	5'-ACCATCATTGGTGTGTCGGTC-3'	
TLR4 R	5'-AGCCAGCAAGAAGCATCAGG-3'	
ICAM1 F	5'-GGCCGGCCAGCTTATACAC-3'	
ICAM1 R	5'-TAGACACTTGAGCTCGGGCA-3'	
MMP2 F	5'-GCGGCGGTCACAGCTACTT-3'	

MMP2 R	5’-CACGCTCTTCAGACTTTGGTTCT-3’	
MMP9 F	5’-AGGCGCTCATGTACCCTATGTAC-3’	
MMP9 R	5’-GCCGTGGCTCAGGTTCA-3’	
IL-1β F	5’-GATGTCTGGTCCATATGAACTG-3’	
IL-1β R	5’-TTGGGATCTACACTCTCCAGC-3’	
IL-6 F	5’-AAATTCGGTACATCCTCGACGGCA-3’	1. 95˚C, 30 s
IL-6 R	5’-AGTGCCTCTTTGCTGCTTTCACAC-3’	2. 56˚C, 30 s
IL-8 F	5’-ATGACTTCCAAGCTGGCCGTGGCT-3’	3. 72˚C, 45 s
IL-8 R	5’-TCTCAGCCCTCTTCAAAAACTTCT-3’	
TNF-α F	5’-GTCAGATCATCTTCTCGAACCCCGA-3’	
TNF-α R	5’-CAGGGCAATGATCCCAAAGTAGA-3’	
iNOS F	5’-CCGAGGCAAACAGCACATTC-3’	1. 95˚C, 30 s
iNOS R	5’-GGTTGGGGGTGTGGTGATGT-3’	2. 60˚C, 120 s
		3. 72˚C, 45 s
**Mouse primers**		
Ef-2 F	5’-GACATCACCAAGGGTGTGCAG-3’	
Ef-2 R	5’-TCAGCACACTGGCATAGAGGC-3’	
Il-1β F	5’-TGCCACCTTTTGACAGTGATG-3’	
Il-1β R	5’-TGATGTGCTGCTGCGAGATT-3’	1. 95˚C, 30 s
Il-1α F	5’-CGCTTGAGTCGGCAAAGAAA-3’	2. 60˚C, 30 s
Il-1α R	5’-AACTGTAGTCTTCGTTTTCACTGT-3’	3. 72˚C, 30 s
Mcpip-1 F	5’-CAGCCTCGACCAGATGTGCC-3’	
Mcpip-1 R	5’-CAGCCGCTCCTCGATGAAGC-3’	
